# Gender Determinants of Vaccination Status in Children: Evidence from a Meta-Ethnographic Systematic Review

**DOI:** 10.1371/journal.pone.0135222

**Published:** 2015-08-28

**Authors:** Sonja Merten, Adriane Martin Hilber, Christina Biaggi, Florence Secula, Xavier Bosch-Capblanch, Pem Namgyal, Joachim Hombach

**Affiliations:** 1 Swiss Tropical and Public Health Institute, Basel, Switzerland; 2 University of Basel, Basel, Switzerland; 3 Initiative for Vaccines Research, World Health Organization, Geneva, Switzerland; McGill University Health Centre, CANADA

## Abstract

Using meta-ethnographic methods, we conducted a systematic review of qualitative research to understand gender-related reasons at individual, family, community and health facility levels why millions of children in low and middle income countries are still not reached by routine vaccination programmes. A systematic search of Medline, Embase, CINAHL, Cochrane Library, ERIC, Anthropological Lit, CSA databases, IBSS, ISI Web of Knowledge, JSTOR, Soc Index and Sociological Abstracts was conducted. Key words were built around the themes of immunization, vaccines, health services, health behaviour, and developing countries. Only papers, which reported on in-depth qualitative data, were retained. Twenty-five qualitative studies, which investigated barriers to routine immunisation, were included in the review. These studies were conducted between 1982 and 2012; eighteen were published after 2000. The studies represent a wide range of low- to middle income countries including some that have well known coverage challenges. We found that women's low social status manifests on every level as a barrier to accessing vaccinations: access to education, income, as well as autonomous decision-making about time and resource allocation were evident barriers. Indirectly, women's lower status made them vulnerable to blame and shame in case of childhood illness, partly reinforcing access problems, but partly increasing women's motivation to use every means to keep their children healthy. Yet in settings where gender discrimination exists most strongly, increasing availability and information may not be enough to reach the under immunised. Programmes must actively be designed to include mitigation measures to facilitate women's access to immunisation services if we hope to improve immunisation coverage. Gender inequality needs to be addressed on structural, community and household levels if the number of unvaccinated children is to substantially decrease.

## Introduction

Routine childhood immunization is fundamental to reducing infant and childhood mortality. The introduction of new vaccines and the ambitious global disease eradication and elimination targets have increased the complexity of vaccination and thereby challenged immunisation programmes and health systems in many countries. Since 1974, when the World Health Organization launched the Expanded Programme on Immunization (EPI) the global coverage of fully immunised children for six preventable childhood diseases—polio, diphtheria, tuberculosis, pertussis (whooping cough), measles and tetanus—rose from 5% to 80% in 1998. Since the millennium turn however coverage rates didn’t improve substantially anymore, and in 2011 global DTP3 coverage (three doses of the diphtheria-tetanus-pertussis combination vaccine) was still not higher than 83% [1]. In 2010, it was estimated that 19.3 million children worldwide were not fully vaccinated [2]. Likewise, the proportion of unvaccinated children–children who never received any dose of a vaccine–remained as high as 28% in some countries. Eight out of the ten countries with the highest rate of unvaccinated children were in the African Region [3].

In 2007 the WHO Strategic Advisory Group of Experts on Immunization (WHO⁄ SAGE) requested a ‘more detailed analysis of children not reached by immunization’ [4]. In response WHO commissioned a series of investigations on prevalence and determinants of children not receiving any dose of vaccine. An analysis of data from 96 countries revealed that low educational status of the caregivers, wealth index, the caregiver’s tetanus toxoid status, and decision-making with respect to treatment-seeking were main determinants of being unvaccinated [3, 5]. Other studies found low maternal education to be associated with only partial vaccination [6, 7], and disparities in immunisation coverage between boys and girls were described [8, 9]. The latter was not a generalised phenomenon, favouring boys in some contexts and girls in others.

Some findings from these studies suggested that caregivers’ gender may be playing a crucial role for accessing vaccination of children. In 2010, following the formulation of the GAVI Alliance (GAVI)’s new gender policy, interest in gendered dimensions of barriers to vaccination provided support for a more in-depth exploration of the issue. The Initiative for Vaccine Research (IVR) therefore commissioned a follow up of the study on children not reached by immunization with a focus on gender aspects. A further, unpublished analysis of the DHS data investigated sex differentials and other possible gender related differences in childhood vaccination status. Since quantitative associations between different maternal factors and child vaccination status cannot fully explain why and how these factors operate, a systematic review of qualitative research focusing on gender aspects of barriers to childhood immunization was conducted in parallel. This paper presents the synthesized findings from this systematic review, which was first presented to WHO in form of a descriptive report, as an update with the permission of the WHO of the 2010 report [10]. The aim of this paper is to offer a contextualized understanding and explanation as to how gender affects access to vaccination in childhood in low- and middle income countries with reflections on how programme planners and managers can better address gendered barriers to immunization in future.

### Theoretical considerations

We refer to gender as a social construct, i.e. as a socially conditioned subjective identity linked to a socially ascribed gender role and position, which is often associated with a lower social status of women, leading to differential health outcomes for women as compared to men [11, 12]. Gendered health differentials, in contrast to biological differences, originate in the different socially constructed risk factors for men and women and in the gendered barriers to accessing healthcare, which affect women differently than men. As children usually gain access to healthcare through women, gendered barriers affect children as well [12]. Access to healthcare can be constrained on different levels, and we hypothesize that some of these barriers are gender-specific. When Sen & Oestlin [13] analysed the role of gender as a social determinant of health, they maintained that gender shapes structural as well as intermediate factors on both the user and the health system side. These considerations framed the rationale for this systematic review, but without a priori structuring the synthesis of the findings.

## Methods

**Fig 1:**
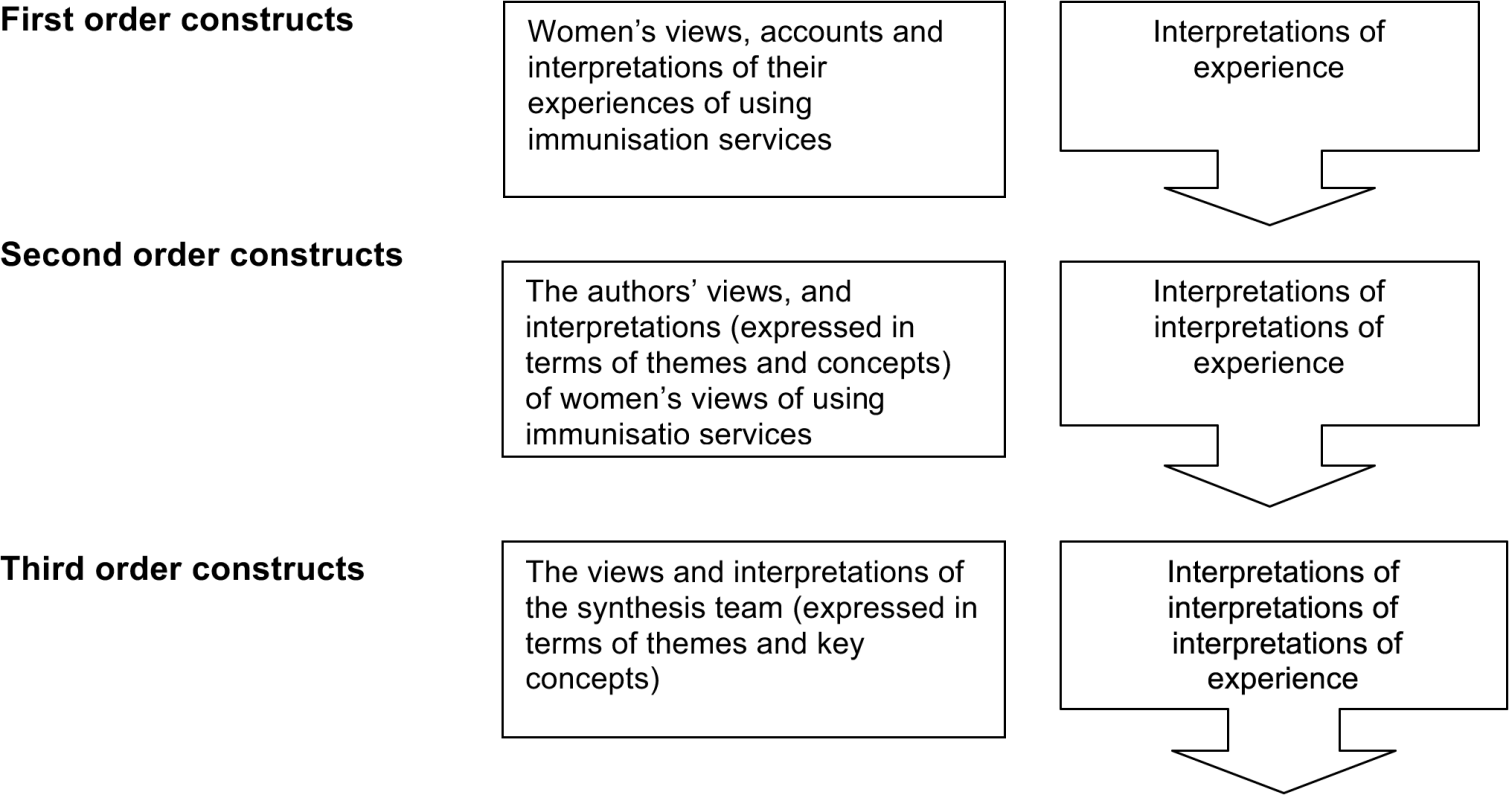
Definition of 1st, 2nd and 3rd order constructs (adapted from Malpass et al. 2009).

This qualitative systematic review using meta-ethnography methods was conducted from October 2009 to June 2012. Meta-ethnography, since first described by Noblit and Hare [14], has proven to be a useful tool to synthesize the evidence from qualitative studies in order to attain a deeper understanding of complex health-related topics [15]. According to Malpass, the systematic reading and interpretation of the text requires the clear distinction between first, second and third-order constructs (Fig 1). First-order constructs refer to original narratives in the reviewed articles that relate to a particular topic. The interpretations of these narratives by the authors of the reviewed articles are considered second-order constructs, while the synthesis of the review team are third-order constructs [16]. Meta-ethnography uses the process of “translation” to synthesise first- and second-order constructs, whereby the findings of a study are examined in relation to other studies for whether they refer to the same concepts (“reciprocal translation”), opposite concepts (“refutational translation”) or can be ordered so that they create a “line of argument” [17]. Meta ethnography, as opposed to a literature review, thus provides a systematic and structured approach to the analysis and synthesis of findings across multiple studies [18].

### Search strategy, inclusion and exclusion criteria

Medline, Embase, CINAHL, Cochrane Library, ERIC, Anthropological Lit, CSA databases, IBSS, ISI Web of Knowledge, JSTOR, Soc Index and Sociological Abstracts were searched. Key words were built around the following themes: immunization, vaccines, health services, health behaviour, and developing countries. There were initially no publication date or geographical restrictions to the literature searches; this was later amended to exclude articles from high-income countries. We restricted the electronic search to English, French, Spanish, Italian, German, and Portuguese language publications (S1 Annex). The search was done on February 17, 2010 and repeated on June 29, 2012. All studies published in peer-reviewed journals from the earliest date of each database up to the cut-off date of the second search (29 June 2012) that contained qualitative methods of data collection and analysis were considered. Grey literature was not included.

Sifting of retrieved citations and coding was done in duplicate independently in three phases; title, abstract and full text. Differences were resolved by consensus. All bibliographies of studies and books that corresponded with the inclusion criteria were scanned to identify additional literature. Studies reporting that the qualitative data was collected through open-ended questions during a structured (quantitative) questionnaires were excluded (N = 293). Full texts of all studies using a qualitative design and analysis method or those for which no abstract was available were retrieved (N = 173).

**Fig 2:**
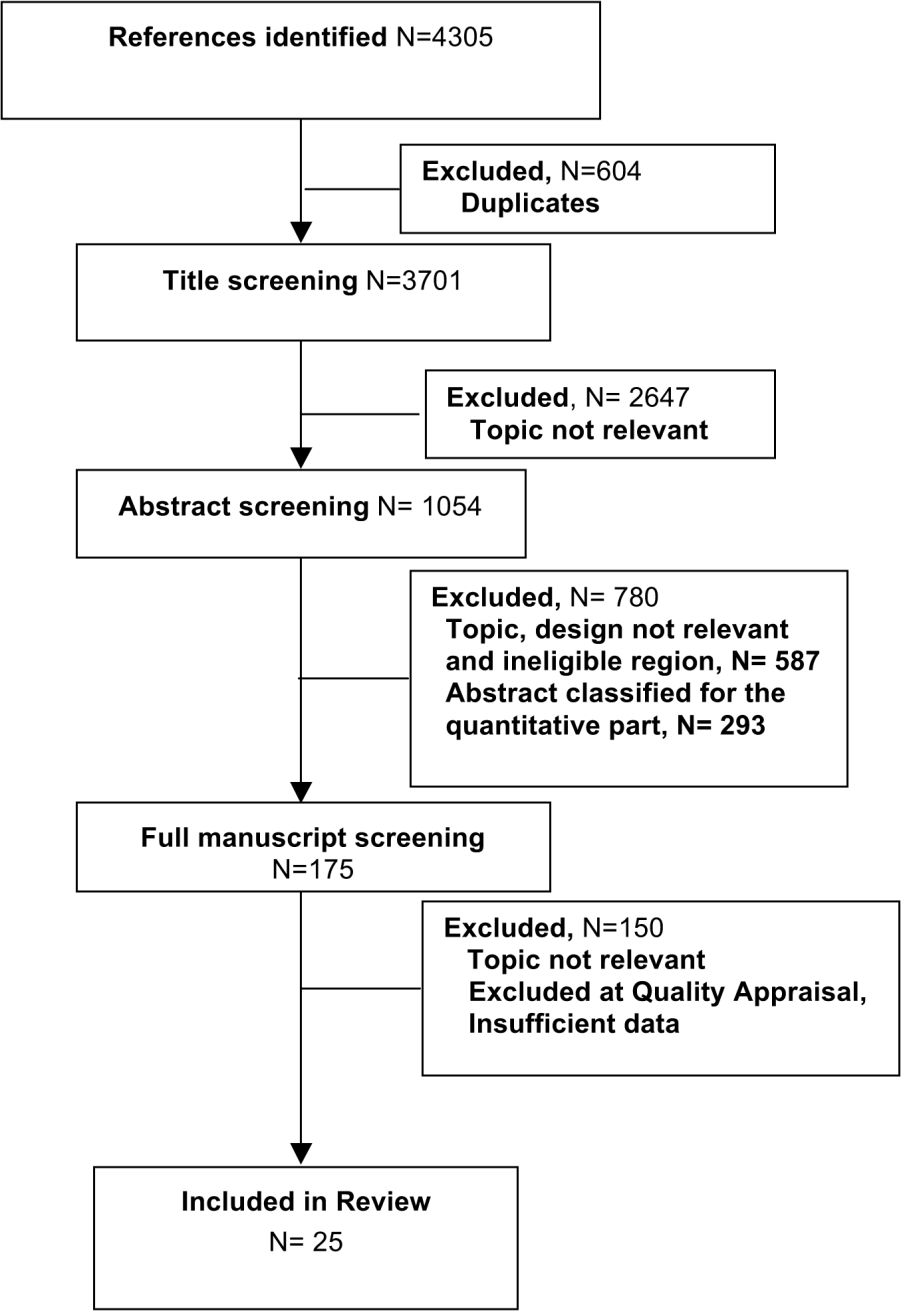
Meta ethnography flowchart.

The quality of studies was assessed by the entire team using published criteria [19] (S1 Annex). Twenty five studies from low and middle income countries which passed the quality appraisal [19] were finally included in this review (Fig 2). All countries ranged in the lower half of the global Human Development Index scale, and all except two were found within the one third of countries with the highest Gender Inequality Index in 2010.

### Data extraction and synthesis method

After the initial coding, all 25 studies were then read by four authors independently who identified and extracted study and sample characteristics together with key findings in the form of quotes, topical themes and concepts. A division was made between the 1st, 2nd and 3rd order constructs during this process in order to first compare the first-order constructs, which are those expressed by research participants themselves and are usually expressed as direct quotes or respondent’s voices summarized by the authors. Second order constructs, which are the interpretation study authors gave to the experiences of the study participants, were then compared in a second step, leading to the third order constructs, which are our interpretation of the identified first and second-order-constructs [16]. During this process, all themes emerging from the papers were consolidated, grouped and labelled in a translation table. As groups of themes were compared with each other, their relation—either as reciprocal, refutational, or as a common line of argument [14]—was explained.

## Results

### Study characteristics

All twenty-five studies included in the review were based on field work done to investigate specific barriers to routine immunisation. The studies were conducted between 1982 and 2012; 18 were published between 2000 and 2012, 5 in the 1990s and 2 in the 1980s. The studies represent a wide range of LMIC: Bolivia (1), Ethiopia (2), India (5), Nigeria (3), Bangladesh (1), Cameroon (1), China (1), Gabon (1), Haiti (1), Kenya (1), Mozambique (2), South Africa (1), Senegal (1), Uganda (2), Togo (1), and Turkey (S2 Annex).

Only the most recent study in Uganda explicitly addressed gendered barriers to immunization. However, gendered practices, gender hierarchies and power dynamics emerge as themes in all of the studies, and most of the interviews that are reported in the papers were conducted exclusively with female respondents.

We distinguished three broad themes in the papers: a) access to vaccination services (including health system, structural, and community- and individual-level factors); b) knowledge and worldview: (effect on) demand of services (including education, health literacy, knowledge claims and experiential knowledge, and non-western medical belief systems); and c) trust in services (including distrust in health systems, politically motivated rumours, and population development policies). The results that follow describe the transversal gender aspects of these themes. The extracted third order constructs are summarized in a table as S3 Annex.

### Access to vaccination services

#### Costs and resource allocation

In low resource settings, if a mother has to take her child for vaccination, she needs to raise the necessary resources. Although immunization services are usually free of charge, almost all formal health services entail indirect costs which are predominantly related to transportation [20–28]. Additional medication, waiting time, and missed opportunities for income generation, which are particularly relevant for single women, poor families and those living in rural areas, are additional constraints mentioned in several studies that are difficult to bear for some mothers [20–28]:

“If I don’t have food, how can I use Uganda shillings 2000 (approximately US$1) for a boda-boda [motorcycle/bicycle transport] to go for immunisation?” (FGD with younger mothers, Uganda) [29]

Such indirect `hidden' costs of service utilization may not be accorded serious consideration in planning and evaluation of health programs. When resources are scarce, women have to reallocate household resources towards meeting everyday needs such as purchasing food. In Haiti, Coreil emphasized that the need to use available resources for basic needs rather than health services affects the economically disadvantaged more directly because poorer families, “at the edge of survival”, tend to depend more on day-to-day acquisition of food [22]. Common in all settings, subsistence takes priority over health related services in general and in particular over immunization especially for poor families material resources may then not be sufficient to take a child to the clinic for immunization, as illustrated by studies in Gabon, Haiti, Nigeria, Turkey, and Uganda [22, 25, 26, 28–30].

In most cases the mother is not alone to take decisions over household spending. Even though responsibility of child health care is usually left to the mother, her socially subordinate role does not provide for the decision-making power on what to spend household resources. Across study regions women depend on other members of the family, primarily the husband but also other relatives such as the mother-in-law for such decisions [22, 25, 26, 28, 31–36]. In Uganda, where men often control family resources, the author concludes that their involvement in child health issues is key for improving access to care; yet the current health system primarily targets mothers as the person responsible for the child’s health care. [29].

#### Feminized tasks and competing obligations

Women in carrying out their daily activities often have to divide their time between maternal (childbearing/rearing responsibilities), domestic, productive (diversified livelihoods activities) and social tasks (attending sick family members, unexpected guests, etc.) [22, 25, 26, 28, 34, 35]. Time constraints limit access to services as mentioned in studies from Bangladesh, China, and Gabon [26, 34, 37]. Familial obligations as in Haiti and Turkey may also impede a mother’s ability to take the child to the immunization session [22, 28].

“The guests had come unexpectedly; you cannot say to the guest to stay at home so that I could take the child to the session, we cannot do such things....' [Mother, Istabul, Turkey] [28]

The gendered intra-household hierarchies often assign women the task of representing the social status of the family externally through her domestic and social role which she cannot challenge without challenging patriarchal hierarchies.

#### Female isolation and lack of self-efficacy

In Turkey where gender norms restrict female mobility in public, women refrain from leaving their homes in order to maintain their reputation and status, or in some cases, out of fear:

“I am a person who had never left the house. I do not have the courage to go from here to there because I am constantly at home. I was also grown up in a village, and also in the village you are always at home, we had come here (to Istanbul) and I am constantly at home here too. If you ask my husband he knows everywhere in Istanbul.... If you tell me to go from here to there, truly I will become terrified. I also tell my husband that if I get lost I probably will cry just like a child.” [Mother, Istanbul, Turkey] [28]

Topuzoglu (2007) comments that “the patriarchal structure of the community required [mothers’] activity to be closely monitored by other members of the family and women were mostly isolated in their neighbourhoods.” In Haiti, Coreil (1994) also reports that some mothers “are just plain shy about being in public and may put off going to a [health] post because of timidity”.

#### Social exclusion

Specific groups of women are particularly disadvantaged. Coreil (1994) described how in Haiti marginalised groups, but also migrants who do not have assistance from friends, family or community, were at risk of not being able to attend health services for immunisation. In these cases, the social network of a mother was too weak to assist her financially or by taking over some of her chores which would allow her to leave home or workplace for some time. In general, women from less-well integrated families are more likely to lack the social connections that could encourage clinic attendance such as social relationships with health staff or socially organized groups of women who attend the clinics together [22, 23, 25, 30, 38].

#### Poverty and shame

Poverty also perpetuates marginalization. Child health status is perceived as a reflection of the capacity of a mother to maintain and nurture her child. A child’s health condition and often represents the entire family’s social status, thus poor mothers may avoid health services for fear public scrutiny. Mothers that cannot dress themselves and their babies in good clothes also avoid health services out of shame [21–28, 35]. Coreil (1994) points out that in Haiti, “To obtain clinic-based services, mothers must enter an arena of public visibility where their performance as caretakers is open to scrutiny. In order to obtain a vaccination for a child, a mother risks being judged in other areas of caretaker competence, such as child nutritional status.” In the words of a young mother:

“[Other mothers] see that the child does not thrive, they prefer to stay at home, instead of going to the vaccination. [20-year-old mother of 1 child, Lambaréné, Gabon] [26]

#### Patronizing counselling

Healthcare providers use blame and disrespectful treatment as a patronizing way of ‘educating’ mothers, as it was observed in many studies from different countries [22, 24, 26, 28, 35, 39]. For example, the lack of privacy in health facilities results in the provider-mother interactions to be shared publicly as one mother from Gabon explains:

“If you come to the MCC, they will not talk to you in private, they will talk to you in front of everybody, to make sure that next time you will not bring it [the baby] like that, you will change….” [20-year-old mother of 1 child, Lambaréné, Gabon] [26]

Disregard of time-allocation problems of clients who face difficult journeys, long waiting times, and the discomfort of crowded waiting areas, as well as the feeling to be rushed when one's turn finally arrived, compromised mothers’ willingness to utilize vaccination services [21, 25, 27, 38–40].

“If you are late, they won’t take you and you have to come back another time. Sometimes you arrive and they have already finished vaccinating. You will be obliged to come back”. [23-year-old mother of 2 children, Lambaréné, Gabon] [26].

#### Psychosocial distress

Living in insecurity creates additional emotional distress which can shift the focus of parents to immediate problem-solving strategies that obtain highest priority, rather than considering long-term health needs. A Turkish mother explains:

“When there are problems at home I cannot think of anything, I even forget about the children. My husband had closed his business, he was not employed for a couple of months, this had a reflection on the children, on our house. I forgot the children, I neglected the children, otherwise it (the shots) would not have been incomplete” [Mother, Istanbul, Turkey] [28].

Emotional distress may limit mother’s ability to act on their own behalf. Furthermore, women are often responsible for the maintenance of the emotional balance in a home, which is especially difficult in absence of social support, and which may further incapacitate mothers to pursue other obligations [28].

#### Discriminatory values: son preference

Only in the study by Li (2004) in China was the sex of the child cited to influence decisions about immunization. In Li’s study in a predominantly patrilineal, rural Chinese context, the ability to give birth to a son determines the mother’s status in the family and society where sons are ascribed a higher value and thus they are given a higher priority than girls in resource allocation terms. Furthermore, as a consequence of China’s one child policy, mothers hid children that were not legitimate according to the one child policy, as well as themselves, from health services. While the one child policy is not a priori gendered, it becomes gendered through the preference of sons, leading to more ‘legitimate’ male children who are more likely to access government health services such as immunization [34].

### Demand for vaccination services

Over the last 3 decades illiteracy has been identified as a major barrier to immunization in all study regions [20, 25, 28, 33–36, 41]. Illiteracy may be a barrier to information as well as an indicator of a lower socioeconomic status and poverty, and has therefore a direct and indirect effect on the demand and utilization of vaccination services. Access to education is usually gendered to the disadvantage of women, but the role of the father’s education has been highlighted as well (here in China) [34].

#### Experiential knowledge and legitimacy of information

Formal education facilitates access to a broad spectrum of health-related information, which is assumed to predicate informed choice. Information about vaccination per se does not however imply its uncritical acceptance or that does it reach its target group. In the early 1980s in Nigeria, for example, half of the mothers with some formal education that were interviewed “did not believe in the vaccine” [41]. A study from Cameroon ten years later suggested that education had no impact on awareness of the existence of immunization programmes nor on beliefs in supernatural causes of diseases [20]. More recent studies in Ethiopia, Kenya, Haiti, India, and Turkey all reveal that a person’s health seeking behaviour is shaped more by “empirical knowledge” derived from the communities’ “long-term experience of the disease itself” rather than from health information provided by the health system [21–23, 27, 28, 41, 42]–yet a finding not limited to low income countries, as some authors state.

Often the source of information is as important as its content as information needs to be credible. Bisht & Coutinho (2000) remind that knowledge production is not isolated from the person who conveys it. Knowledge claims made by health professionals in Kenya “may come into conflict with the existing knowledge of the community”. For example for young mothers, elderly women may be a more authoritative and reliable source of knowledge and information than health professionals [42]–emphasizing the gendered trans-generational flow of cultural inheritance. Political and religious authorities in Togo, Nigeria and India [25, 36, 39], teachers (Nigeria), and the media (India) [33, 39] are alternative sources of information and may formulate conflicting positions towards vaccinations which are legitimate in the respective settings.

#### Concepts of prevention and protection

Anthropological literature has described a wide range of practices to address perceived health threats and to protect a child, including the use of traditional medicines, magic, or prayers and other religious activities [21, 23, 32, 35, 36, 41, 42]. Yet the notion of a preventive health measure targeting a single disease as it is the case with vaccination may be unfamiliar in some medical belief systems. Protecting the child from danger constitutes an essential part of maternal child care in all study regions. In some cases protective measures were aimed at preventing any type of misfortune, and vaccines were considered accordingly as “something good for health” based on beliefs that it helps to build strength and improve a child’s health broadly, or that if a disease develops, it would do so only mildly for the child who has been vaccinated and is now strong [24, 27, 28, 32, 36, 41, 42]:

“Vaccines build up the strength, like Ravita’s (her daughter) cough was cured after she was given the injection (vaccine)…” [Mother in Surat, India] [42].

Especially in more remote settings where biomedical information is less available such as among the indigenous populations of Bolivia where one of the studies was conducted, the way in which biomedical prevention and treatment works may not be understood [23–25, 33, 35, 36, 41, 42].

#### Witchcraft/sorcery as social dimension of illness

Several authors further report that vaccine preventable diseases were believed to be caused by a range of magico-religious factors such as curses or sorcery [20, 21, 23, 27, 33, 35, 36, 41, 42]. Vaccines in such cases might be considered insufficient [25, 32, 42] to ward off danger. Bastien (1995) emphasizes that perceived supernatural causes of illness among the Bolivian indigenous populations should not be regarded as solely due to limited health literacy but rather as interwoven with the social dimension of causal perceptions. He argues that witchcraft accusations are sometimes strategically used to act against persons who are suspected of manipulating and challenging social hierarchies, as “a frequent means of revenge and retaliation”. Thus control of sorcery is critical for social control, and precisely this need for social control makes it difficult to reject sorcery as a cause of illness. Suspecting a supernatural cause of illness is a powerful tool to enforce control over others, and giving up such beliefs would thus imply giving up an important means of control.

According to Bastien (1995) witchcraft as a power discourse is highly gendered: “blame is imputed on the mother for having done something to inflict sorcery upon her child”, which he considers “a way that… men can use to castigate and control women”. Bastien links such gendered perceptions to colonial influences, such as beliefs regarding women as less able to control themselves and as a harbour of evil forces dangerous to the human kind [35].

### Trust in vaccination services

Health systems and public health interventions may be viewed with suspicion if the set priorities do not meet local needs. Health interventions may be perceived as a threat if governments and their collaborating agencies are not trusted. As a consequence resistance to vaccination can signify and trigger political resistance.

#### Resistance to vaccination

Studies in Bolivia, Uganda, India, Nigeria and Turkey all described active resistance to vaccination campaigns [28, 33, 35, 43, 44]. Collective experiences with the historical past were important in these examples. In settings where people experienced coercive population control (India) or colonial health programs in Africa, people were wary. In the example of Nigeria in 2006, fears were raised through rumours of sterilization and genocide so that people were reluctant to vaccinate their children:

“No, I don’t allow the people to do polio vaccination for my children in the house because there is a problem in it, such as that European people want us to reduce our numbers, to stop us from giving birth. And we are looking for medicine in the hospital to give to our children and we can’t get it but this one, they are following us to our houses to give it. I don’t trust this polio vaccine.” [Farmer, Samaru, Nigeria; September 2005] [33]

Renne reports that in Northern Nigeria people questioned the motivation for a polio eradication program that appeared to draw resources away from primary health care. When other basic primary health care services were neither available, nor free of charge, explanations that the authorities were simply interested in the health of the children are difficult to believe [21, 24–26, 30, 33, 45].

Resistance to vaccination reflects the gendered nature of a politicized immunization discourse. When politically based rumours circulate, men get engaged in immunization, as an Indian woman points out:

"Our men keep talking about polio drops, and every time there is something new about it. Sometimes it's about sterility sometimes about pig's blood sometimes about conspiracy against Muslims." [Mother-Muslim, Dingarpur, India] [30].

Chaturvedi (2009) reports in her study in India that due to the wish to keep their children healthy many mothers finally decided to comply with immunization recommendations despite initial resistance [30]. Babirye (2011) observed in her Ugandan case study that distrust toward vaccinations was mainly purported by men, while some women explicitly encouraged others to disregard their husband’s refusal to vaccinate. Strikingly, men’s views were predominantly represented in research when immunization had become a political issue.

## Synthesis and Discussion

**Fig 3:**
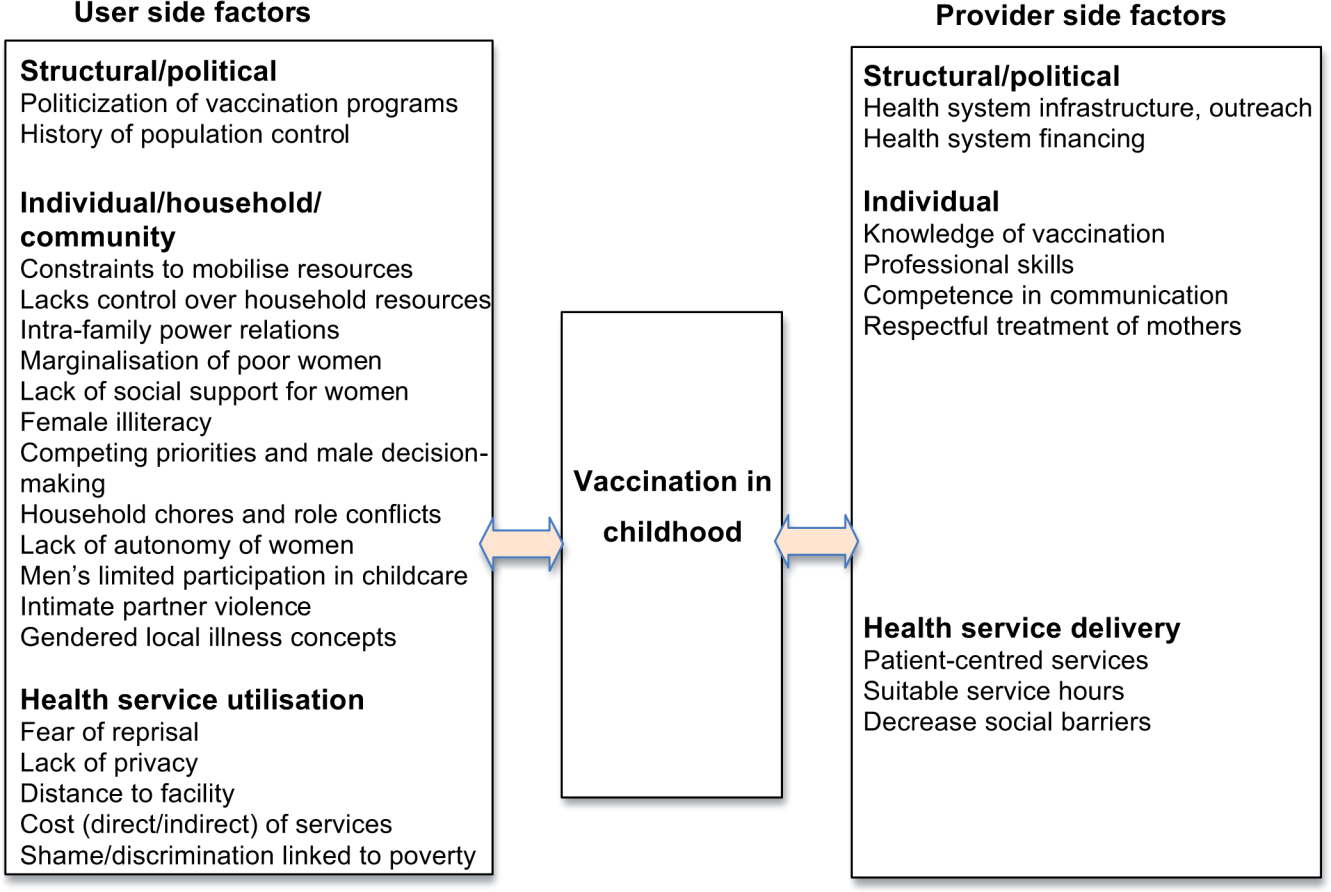
Gendered factors influencing vaccination in childhood–overview over the main second-order constructs.

**Fig 4:**
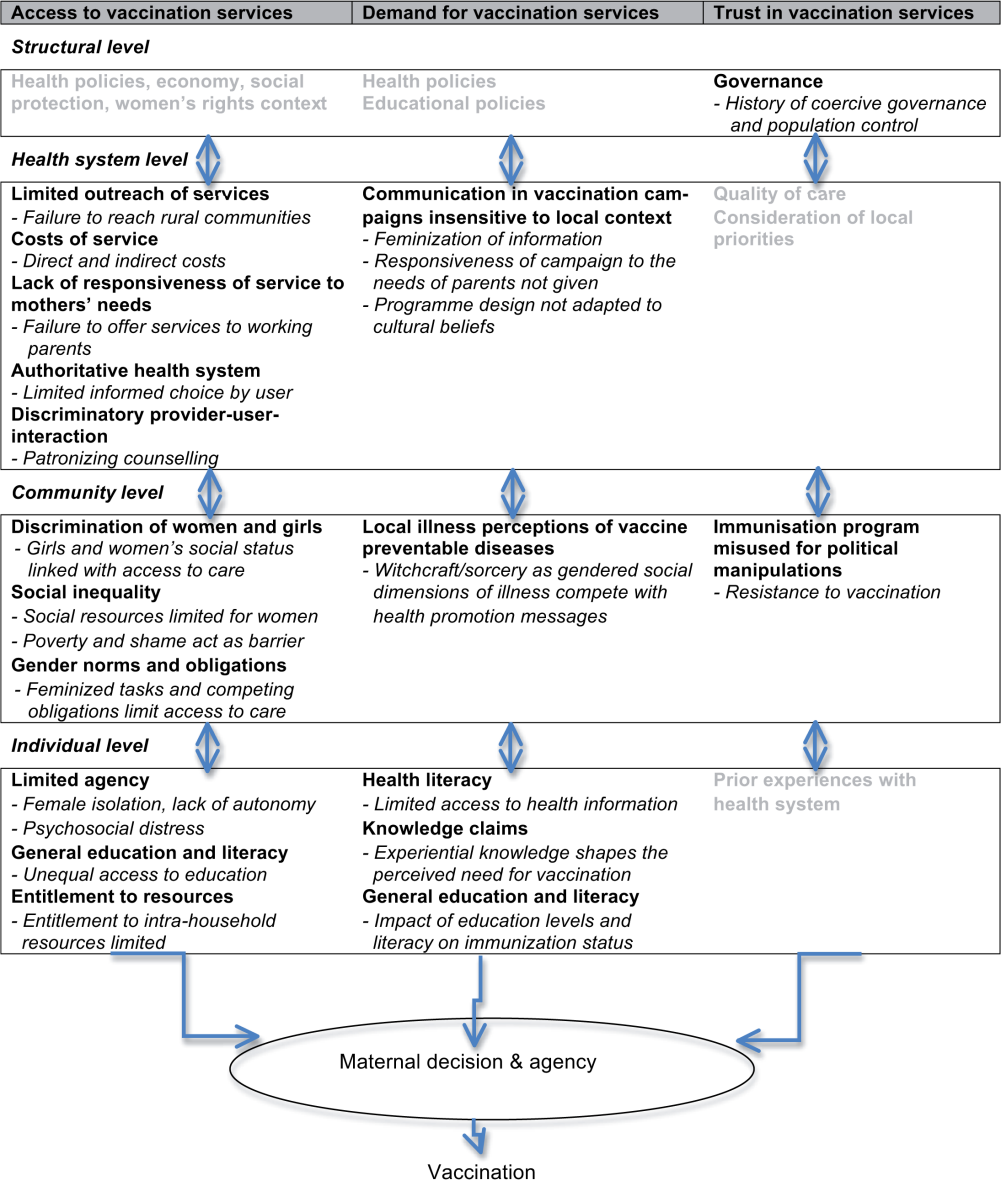
Third-level constructs of gendered factors influencing vaccination in childhood.

The review of gendered facets of barriers to immunization revealed many gendered factors that impede uptake of vaccination (Fig 3). General access-related barriers such as inherent limitations of vaccination services, caregiver vulnerability and parental attitudes and knowledge had been identified before, however without a focus on the structuring role of gender [46–49]. High gender inequality characterized most of the study settings: all except two were within a third of countries with the highest Gender Inequality Index in 2010. Gender as a social construct affects access to healthcare, demand for health interventions, and trust in the health system, through gendered structural determinants and associated gendered vulnerabilities, discriminatory values, norms, and practices, and biases in health systems [13]. This review showed that gender inequality is relevant on the structural, health system, community, and individual level (Fig 4).

Theories of access to healthcare, often referring to Penchansky and Thomas’ five dimensions of access to healthcare, which comprise availability, accessibility, accommodation, affordability, and acceptability of services, usually have a strong focus on health system factors in contrast to theories of treatment seeking behaviour [50]. Access constraints are however found on both the user and on the health system side. Waiting times when seeking care in a health facility is an example of a provider-side factor, which also constrains women’s options to use the service. Women often have only a limited time available to spend outside their home, and they are expected to meet the needs of other household members as well. Health system constraints may thus reinforce gendered access constraints caused by women’s limited autonomy.

### Constrained agency: social norms and economic inequality

Women’s agency is shaped and constrained by a variety of structural and local processes. Agency is here understood as a woman’s capacity for action on her own behalf [51]. On the one hand, women often have a lower educational status, less income, less decision-making power over household resources but greater responsibility for childcare and children’s health [52–54]. In low-and middle-income countries many women are struggling to mobilize the necessary means for transport and health services needed to take a child for vaccination. In presence of competing needs resources may not be primarily allocated to preventative and thus not urgent interventions like vaccinations. On the other hand child health services are often biased towards the assumption that women alone are responsible for child healthcare, neglecting their restricted access to resources and decision-making power [29]. These compromising conditions are a consequence of and reinforced by local social norms and values [22, 26, 35]. Gendered responsibilities and blame often compound on women who may be publicly scolded because of their inability to overcome structural constraints. Particularly striking were the accounts of impoverished women who were publicly accused by health professionals of neglecting their children, a humiliation reinforcing their already low social status and general vulnerability [22, 26]. In contrast, the right of the father to deny a mother the resources to take a child for immunization is not challenged. Health systems may perpetuate these societal values by addressing primarily mothers as the key actor for child healthcare. It neglects women’s realities as responsible for household production. There has been an inclination to assume that women who work in the informal sector or for the family have free time, which reflects the lower importance assigned to their work [55]. As a result, the multiple roles of women (productive, maternal and social role) are often not taken into account in the design of vaccinations programmes. Social, time, and missed opportunity costs are borne almost exclusively by women. Women continue to be responsible for maintenance of family and household: they spend many more hours per day gathering water or fuel, preparing food or taking care of children and/or visiting family or helping neighbours [56, 57]. Time constraints due to competing obligations pose significant access barriers for women and children under their care to health services. This is aggravated if a woman’s and her children’s needs are subordinated to the needs of other household members who control much of a woman’s time or restrict her ability to decide freely over how to set priorities [52–54].

The predisposition to assume that women as main caregivers should be primarily responsible for taking a child for immunization falls especially short in contexts with high levels of gender inequality.

There has been a growing demand for more involvement of fathers in child health programs [52, 54] particularly with respect to vaccines [29, 58]. The current health system context is however not favourable to fathers. Health facilities are perceived as feminized spaces. In the context of antenatal care for example it has been shown that many men are reluctant to accompany their wives to the health facility, which is considered as ‘designed for women only’ [59]. Further consideration should be given to strategies to engage men in child healthcare with attention that they can jeopardize women’s empowerment and role within the family. Male involvement increases the legitimacy of male decision-making power over family matters, which may reinforce gender inequalities and compromise beneficial aspects of male involvement in healthcare [54].

### Maternal tactics

It is well known that knowledge that maternal education influences immunization coverage. A paradox emerges however when belief in supernatural causes of illness are not limited to the least educated [20]. Local values and belief systems may provide tactical options for women to negotiate responsibilities for child illness and death. Ethnographic studies on treatment-seeking suggest that mothers sometimes refer to supernatural explanations of illness to avert allegations of insufficiency and failure even if they are sufficiently informed about adequate treatment procedures [54, 60, 61]. However, indigenous belief systems, may also blame mothers. Bastien for example pointed out that in Bolivia women were considered responsible for attracting the anger or jealousy of a person who was said to have attacked them through magic, a curse, or witchcraft [35]. In such a situation women are under enormous pressure to deflect accusations of harming their children through ‘wrong’ behaviour and have little bargaining power. Similarly tactical women may act in contexts where they are not expected to take decisions against authoritative members of the community, such as their husband, a senior female relative or in-law, or a religious leader. In this case women do not necessarily prioritize information from health professionals as ‘experts’ if the expert has a lower social position [36, 42].

### Gender aspects of politically motivated resistance to vaccination

Childhood immunization campaigns have been contested repeatedly and served as a surrogate for the conflicts of local political opponents. Broad resistance to vaccines was observed in some countries such as India, China, and Nigeria, linked to rumours on infertility, which is reminiscent of coercive family planning policies that may bring back fear and anger experienced in the past under oppressive policies [30, 33, 34, 49]. While threats are perceived to concern a group as a whole (e.g. all Muslims), they are indicative of other power struggles waged in these settings including men’s control of women’s fertility. Often politically motivated resistance to immunisation is summoned by male leaders but must be enacted by women when they refuse to immunise their children. Women and children’s constrained right to health is may be a precondition for resistance against immunization. Chaturvedi (2009) and Babirye (2011) speaking of India and Uganda both show that some women began vaccinating their children despite opposition expressed by male leaders or husbands, sometimes motivating other women to disregard men’s positions as well.

Most gendered aspects of barriers are rooted in gendered norms and rules that shape perceptions, values, expectations, rights, feelings, and behaviours (Fig 4). Empowerment of women to increase their agency is still urgently needed and is likely to improve child healthcare, including immunization [62]. Changes at the level of social norms and regulations are indispensable [13]. In many societies health-related decision-making is a collective process and empowering mothers requires strengthening her position and bargaining power in the gendered and generational dynamics of the group, and to improve women’s status and rights in her community. Immunisation services play out in societies and communities. More availability of services in their current format and more education and information will not necessarily reach the under immunised sufficiently. In settings where gender discrimination exists most strongly, programmes must actively be designed to include mitigation measures to facilitate women’s access to immunisation services if we hope to reach the currently under served.

### Limitations of the study

Most qualitative studies included in this review were conducted in areas where vaccination delivery (e.g. campaigns and / or routine vaccination) faced considerable resistance and lack of uptake, and information was needed to address these problems. Two vaccine acceptability studies were conducted in order to elucidate the socio-cultural context in which vaccination would take place [35, 45]. Only one study was conducted to investigate gender-specific barriers in particular [29], and only one obtained information from fathers [33]; the rest relied on responses given by children's mothers. Furthermore, the studies are not resource-oriented in the sense that women’s ability to mobilise support are not explicitly addressed; they remain barrier-oriented and have a strong focus on health system management; this may however not be sufficient to overcome structural and value-based constraints.

Meta-Ethnography, if based on published literature only, has some limitations in itself as well. As we have no access to the primary data, only what the authors of the included articles considered relevant enters our synthesis. As such, the synthesis is not necessarily comprehensive.

Despite these limitations and despite heterogeneity of contexts and time, the studies implicitly contained information about the gendered nature of child immunization programs. A strength of this study is that it synthesizes the complexity of the different layers of the influence of gender on immunization in childhood, and it could show that the mechanisms, how gender inequality contributes to underimmunization, are more complex than commonly assumed. A woman’s often lower educational status, for example, which is a known determinant of vaccination status, may not only reflect a lesser knowledge of the benefits of vaccination. A lower education also constrains a woman’s access to material resources and may lead to discrimination in the health facility, among other issues. Where women are restricted in their autonomy due to gender norms that value men’s interests higher than women’s, women may be overburdened in order to serve the family, and health education alone may not offset these constraints. The study thus shows as well that programs, which build on Western assumptions of gender roles and female empowerment, may miss the needs of women in other societies and instead may add an additional burden on women by increasing expectations on their tasks. There is an urgent need for a gender sensitive assessment of different approaches, which aim at increasing uptake of services.

## Conclusions

Maternal and child health, including vaccinations, are a key component of the primary health care approach. To the extent that vaccination targets mothers rather than parents, vaccination programmes can reflect and therefore reinforce gender and social dynamics found in the communities where they operate. Persisting gender inequality negatively affects women’s access to vaccination through their limited decision-making power over resources and constrained agency. Many dimensions of inequality have a formalized correlate in law and policy, which will continue to restrict women’s access to health services. Continuous efforts to overcome underlying structural gender inequality are still needed.

Improvements in vaccination coverage may be obtained through fathers’ and communities’ involvement in vaccination initiatives. The transformative power of men’s involvement through innovative programming can help to address power inequalities resulting from gender bias [13]. By targeting communities and parents in programme strategies, and not women alone, responsibilities for child health can be expanded from the mother alone to the parents and the broader community for better health outcomes for the child.

Power politics of immunisation are played out in some regions by political and religious leaders for political gain. In such cases, the cost is again borne largely by women as caretakers and their un-immunised children on the frontlines of resistance. Increasing coverage requires acknowledgment of the unequal power dynamic that exists within the service setting, the politicization of health care services, and the marginalized position of women in these settings. This study shows that overcoming compounded gendered barriers to children’s immunisation requires expanding the dialogue around vaccination beyond the mother as primary caretaker to extended family, fathers and communities. When immunisation of children becomes a family and community responsibility, more progress can be made.

## Supporting Information

S1 PRISMA Checklist (DOC)

S1 Annex Search strategy and SOP. (DOC)

S2 Annex Study characteristics table. (DOC)

S3 Annex Translation table. (DOC)
