# Supplementary material for: Gender Determinants of Vaccination Status in Children: Evidence from a Meta-Ethnographic Systematic Review
Source: PLoS One. 2015 Aug 28;10(8):e0135222. doi: 10.1371/journal.pone.0135222 (PMC4552892; doi:10.1371/journal.pone.0135222)
Supplement: S1 Annex — (DOC) [file pone.0135222.s002.doc]

**S1 Annex - Search strategy and SOP: *Immunization and Gender Systematic Qualitative Search***

1. **General information**

**Research Question:** How do gender factors contribute to the explanation why some children fail to receive all EPI vaccination through the routine immunization program in developing countries (epidemiology of unvaccinated children) ?

**Design:** Systematic review of published literature (articles and books)

**Time period:** not restricted

**Initial search criteria:** Broad search to encompass all aspects of immunization service delivery and acceptance of immunization in developing countries. Developing countries defined according to UN’s listing of low and middle income countries

**Databases used:**

Medline + (Ovid)

EMBASE

JSTOR

**Sociological Abstracts**

**Soc Serv Abs**

ERIC

Cochrane

*CINAHL*

IBSS

CSA databases

Soc Index

Anthropological Lit

Wiley Interscience

ISI Web of Knowledge (Web of Science)

**Languages:**

English

French

Spanish

Portuguese

Italian

**Key Words:**

1) exp Immunization/ or exp vaccines/ or vaccine* or vaccinat* or immuniz* or immunis*

2) Countries (see below)

3) Service* or intervention*

4) Health Seeking behavio* or access

**Limits:**

Children >5

Humans (no animals)

Articles, reviews, meta analysis, Cochrane, books

*Veterinary Medicine/ or exp *Rabies/ or exp *Rabies virus/ or exp *Avian Influenza/or exp *Influenza /or exp *Human Immunodeficiency Virus/ or (immunolog* or serolog*).ti

1. **Search strategy**

**A. Initial identification of published literature**

**1) MEDLINE+ (17,751)**

**exp Immunization/** or **exp vaccines/** or vaccine* or vaccinat* or immuniz* or immunis*

AND

**exp Developing Countries/** or ((develop* or low income or low-income or lower income or lower-income or middle income or middle-income) adj5 (country or countries)) or Afghan* or Bangladesh* or Benin or Burkina Faso or Burkinabe or Burundi* or Cambodia* or African or Chad or Chadian or Comoros or Cormoran or Congo* or Cote d'Ivoire or Ivorian or Eritrea* or Ethiopia* or Gambia or Ghana* or Guinea* or Guinea-Bissau* or Haiti* or Kenya* or Korea* or Kyrgyz or Kirghiz or Lao or Laotian or Liberia* or Madagascar or Malagasy or Malawi* or Mali or Malian or Mauritania* or Mozambi* or Burma or Burmese or Myanmar* or Nepal or Nepalese or Niger or Nigeri* or Pakistan* or Papua New Guinea* or Rwanda* or Sao Tome* or Principe or Senegal* or Sierra Leone* or Solomon Island* or Somali* or Tajik* or Tadzhik or Tanzania* or Togo or Togolese or Uganda* or Uzbek* or Vietnam* or Yemen* or Zambia* or Zimbabwe* or Albania* or Algeria* or Angola* or Armenia* or Azerbaijan* or Bhutan* or Bolivia* or Bosnia* or Herzegovina* or Cameroon* or Cape Verd* or China or Chinese or Colombia* or Congo* or Djibouti or Dominican or Ecuador* or Egypt* or El Salvador or Salvadoran or Georgia* or Guatemala* or Guyana or Hondura* or India or Indonesia* or Iran or Iranian or Iraq* or Jordan* or Kiribati or Lesotho or Mosotho or Basotho or Macedonia* or Maldives or Maldivan or Marshall Islands or Marshallese or Micronesia* or Moldova* or Mongolia* or Morocc* or Namibia* or Nicaragua* or Paraguay* or Peru or Peruvian or Philippines or Filipino or Samoa* or Sri Lanka* or Sudan or Sudanese or Swaziland or Swazi or Syria* or Thailand or Thai or Timor-Leste or Tonga* or Tunisia* or Turkmen* or Ukrain* or Vanuatu or Ni-Vanuatu or West Bank or Gaza or American Samoa or Argentin* or Belarus* or Belize* or Botswana or Motswana or Batswana or Brazil* or Bulgaria* or Chile or Chilean or Costa Rica* or Croatia or Croat or Cuba or Cuban or Dominica or Fiji* or Gabon or Gabonese or Grenada or Grenadian or Grenadan or Jamaica* or Kazakhstan* or Latvia* or Lebanon or Lebanese or Libya* or Lithuania* or Malaysia* or Mauritius or Mauritian or Mayotte or Mexic* or Montenegro or Palau* or Panama* or Poland or Pole or Polish or Romania* or Russian or Serbia* or Montenegrin or Seychell* or South Africa* or "Kitts and Nevis" or Kittian or Nevisian or "St. Lucia" or Saint Lucia* or "St. Vincent" or Saint Vincent or Grenadines or Suriname* or Turkey or Turk or Uruguay* or Venezuela*

exclude:

animal studies

**exp *Veterinary Medicine/ or exp *Rabies/ or exp *Rabies virus/ or exp *Influenza in Birds/ or exp *HIV/** or (immunolog* or serolog*).ti

**2) EMBASE 1980-current (13,228)**

exp Immunization/ or exp vaccine/ or (vaccine* or vaccinat* or immuniz* or immunis*)

AND

exp Developing Country/ or ((develop* or low income or low-income or lower income or lower-income or middle income or middle-income) adj5 (country or countries)) or (Afghan* or Bangladesh* or Benin or Burkina Faso or Burkinabe or Burundi* or Cambodia* or African or Chad or Chadian or Comoros or Cormoran or Congo* or Cote d'Ivoire or Ivorian or Eritrea* or Ethiopia* or Gambia or Ghana* or Guinea* or Guinea-Bissau* or Haiti* or Kenya* or Korea* or Kyrgyz or Kirghiz or Lao or Laotian or Liberia* or Madagascar or Malagasy or Malawi* or Mali or Malian or Mauritania* or Mozambi* or Burma or Burmese or Myanmar* or Nepal or Nepalese or Niger or Nigeri* or Pakistan* or Papua New Guinea* or Rwanda* or Sao Tome* or Principe or Senegal* or Sierra Leone* or Solomon Island* or Somali* or Tajik* or Tadzhik or Tanzania* or Togo or Togolese or Uganda* or Uzbek* or Vietnam* or Yemen* or Zambia* or Zimbabwe* or Albania* or Algeria* or Angola* or Armenia* or Azerbaijan* or Bhutan* or Bolivia* or Bosnia* or Herzegovina* or Cameroon* or Cape Verd* or China or Chinese or Colombia* or Congo* or Djibouti or Dominican or Ecuador* or Egypt* or El Salvador or Salvadoran or Georgia* or Guatemala* or Guyana or Hondura* or India or Indonesia* or Iran or Iranian or Iraq* or Jordan* or Kiribati or Lesotho or Mosotho or Basotho or Macedonia* or Maldives or Maldivan or Marshall Islands or Marshallese or Micronesia* or Moldova* or Mongolia* or Morocc* or Namibia* or Nicaragua* or Paraguay* or Peru or Peruvian or Philippines or Filipino or Samoa* or Sri Lanka* or Sudan or Sudanese or Swaziland or Swazi or Syria* or Thailand or Thai or Timor-Leste or Tonga* or Tunisia* or Turkmen* or Ukrain* or Vanuatu or Ni-Vanuatu or West Bank or Gaza or American Samoa or Argentin* or Belarus* or Belize* or Botswana or Motswana or Batswana or Brazil* or Bulgaria* or Chile or Chilean or Costa Rica* or Croatia or Croat or Cuba or Cuban or Dominica or Fiji* or Gabon or Gabonese or Grenada or Grenadian or Grenadan or Jamaica* or Kazakhstan* or Latvia* or Lebanon or Lebanese or Libya* or Lithuania* or Malaysia* or Mauritius or Mauritian or Mayotte or Mexic* or Montenegro or Palau* or Panama* or Poland or Pole or Polish or Romania* or Russian or Serbia* or Montenegrin or Seychell* or South Africa* or "Kitts and Nevis" or Kittian or Nevisian or "St. Lucia" or Saint Lucia* or "St. Vincent" or Saint Vincent or Grenadines or Suriname* or Turkey or Turk or Uruguay* or Venezuela*).ti,ab

***NOTE: because EMBASE has journals more likely to have been published in other countries, I limited the "country" part of the search to title and abstract to avoid author affiliation, publisher, etc.*

exclude:

animal studies

exp *Veterinary Medicine/ or exp *Rabies/ or exp *Rabies virus/ or exp *Avian Influenza/ or exp *Human Immunodeficiency Virus/ or (immunolog* or serolog*).ti

**3) Sociological Abstracts (CSA) (217)**

**4) Social Services Abstracts 1979-current (CSA) (126)**

**5) ERIC (CSA) (126)**

DE="immunization programs" or DE="vaccination" or KW=(vaccine* or vaccinat* or immuniz* or immunis*)

AND

DE=("developing countries") or KW=((develop* or low income or low-income or lower income or lower-income or middle income or middle-income) within 5 (country or countries)) or KW=(Afghan* or Bangladesh* or Benin or Burkina Faso or Burkinabe or Burundi* or Cambodia* or African or Chad or Chadian or Comoros or Cormoran or Congo* or Cote d'Ivoire or Ivorian or Eritrea* or Ethiopia* or Gambia or Ghana* or Guinea* or Guinea-Bissau* or Haiti* or Kenya* or Korea* or Kyrgyz or Kirghiz or Lao or Laotian or Liberia* or Madagascar or Malagasy or Malawi* or Mali or Malian or Mauritania* or Mozambi* or Burma or Burmese or Myanmar* or Nepal or Nepalese or Niger or Nigeri* or Pakistan* or Papua New Guinea* or Rwanda* or Sao Tome* or Principe or Senegal* or Sierra Leone* or Solomon Island* or Somali* or Tajik* or Tadzhik or Tanzania* or Togo or Togolese or Uganda* or Uzbek* or Vietnam* or Yemen* or Zambia* or Zimbabwe* or Albania* or Algeria* or Angola* or Armenia* or Azerbaijan* or Bhutan* or Bolivia* or Bosnia* or Herzegovina* or Cameroon* or Cape Verd* or China or Chinese or Colombia* or Congo* or Djibouti or Dominican or Ecuador* or Egypt* or El Salvador or Salvadoran or Georgia* or Guatemala* or Guyana or Hondura* or India or Indonesia* or Iran or Iranian or Iraq* or Jordan* or Kiribati or Lesotho or Mosotho or Basotho or Macedonia* or Maldives or Maldivan or Marshall Islands or Marshallese or Micronesia* or Moldova* or Mongolia* or Morocc* or Namibia* or Nicaragua* or Paraguay* or Peru or Peruvian or Philippines or Filipino or Samoa* or Sri Lanka* or Sudan or Sudanese or Swaziland or Swazi or Syria* or Thailand or Thai or Timor-Leste or Tonga* or Tunisia* or Turkmen* or Ukrain* or Vanuatu or Ni-Vanuatu or West Bank or Gaza or American Samoa or Argentin* or Belarus* or Belize* or Botswana or Motswana or Batswana or Brazil* or Bulgaria* or Chile or Chilean or Costa Rica* or Croatia or Croat or Cuba or Cuban or Dominica or Fiji* or Gabon or Gabonese or Grenada or Grenadian or Grenadan or Jamaica* or Kazakhstan* or Latvia* or Lebanon or Lebanese or Libya* or Lithuania* or Malaysia* or Mauritius or Mauritian or Mayotte or Mexic* or Montenegro or Palau* or Panama* or Poland or Pole or Polish or Romania* or Russian or Serbia* or Montenegrin or Seychell* or South Africa* or "Kitts and Nevis" or Kittian or Nevisian or "St. Lucia" or Saint Lucia* or "St. Vincent" or Saint Vincent or Grenadines or Suriname* or Turkey or Turk or Uruguay* or Venezuela*)

exclude:

TI=(immunolog* or serolog*)

**6) COCHRANE (2039)**

exp Immunization/ or exp vaccines/ or vaccine* or vaccinat* or immuniz* or immunis*

AND

exp Developing Countries/ or ((develop* or low income or low-income or lower income or lower-income or middle income or middle-income) near/5 (country or countries)) or Afghan* or Bangladesh* or Benin or Burkina Faso or Burkinabe or Burundi* or Cambodia* or African or Chad or Chadian or Comoros or Cormoran or Congo* or Cote d'Ivoire or Ivorian or Eritrea* or Ethiopia* or Gambia or Ghana* or Guinea* or Guinea-Bissau* or Haiti* or Kenya* or Korea* or Kyrgyz or Kirghiz or Lao or Laotian or Liberia* or Madagascar or Malagasy or Malawi* or Mali or Malian or Mauritania* or Mozambi* or Burma or Burmese or Myanmar* or Nepal or Nepalese or Niger or Nigeri* or Pakistan* or Papua New Guinea* or Rwanda* or Sao Tome* or Principe or Senegal* or Sierra Leone* or Solomon Island* or Somali* or Tajik* or Tadzhik or Tanzania* or Togo or Togolese or Uganda* or Uzbek* or Vietnam* or Yemen* or Zambia* or Zimbabwe* or Albania* or Algeria* or Angola* or Armenia* or Azerbaijan* or Bhutan* or Bolivia* or Bosnia* or Herzegovina* or Cameroon* or Cape Verd* or China or Chinese or Colombia* or Congo* or Djibouti or Dominican or Ecuador* or Egypt* or El Salvador or Salvadoran or Georgia* or Guatemala* or Guyana or Hondura* or India or Indonesia* or Iran or Iranian or Iraq* or Jordan* or Kiribati or Lesotho or Mosotho or Basotho or Macedonia* or Maldives or Maldivan or Marshall Islands or Marshallese or Micronesia* or Moldova* or Mongolia* or Morocc* or Namibia* or Nicaragua* or Paraguay* or Peru or Peruvian or Philippines or Filipino or Samoa* or Sri Lanka* or Sudan or Sudanese or Swaziland or Swazi or Syria* or Thailand or Thai or Timor-Leste or Tonga* or Tunisia* or Turkmen* or Ukrain* or Vanuatu or Ni-Vanuatu or West Bank or Gaza or American Samoa or Argentin* or Belarus* or Belize* or Botswana or Motswana or Batswana or Brazil* or Bulgaria* or Chile or Chilean or Costa Rica* or Croatia or Croat or Cuba or Cuban or Dominica or Fiji* or Gabon or Gabonese or Grenada or Grenadian or Grenadan or Jamaica* or Kazakhstan* or Latvia* or Lebanon or Lebanese or Libya* or Lithuania* or Malaysia* or Mauritius or Mauritian or Mayotte or Mexic* or Montenegro or Palau* or Panama* or Poland or Pole or Polish or Romania* or Russian or Serbia* or Montenegrin or Seychell* or South Africa* or "Kitts and Nevis" or Kittian or Nevisian or "St. Lucia" or Saint Lucia* or "St. Vincent" or Saint Vincent or Grenadines or Suriname* or Turkey or Turk or Uruguay* or Venezuela*

exclude:

exp *Veterinary Medicine/ or exp *Rabies/ or exp *Rabies virus/ or exp *Influenza in Birds/ or exp *HIV/ or (immunolog* or serolog*):ti

**7) Web of Science (11866)**

TS=(vaccine* or vaccinat* or immuniz* or immunis*)

AND

TS=((develop* or low income or low-income or lower income or lower-income or middle income or middle-income) SAME (country or countries)) or *need to do in chunks*

TS=(

Afghan* or Bangladesh* or Benin or Burkina Faso or Burkinabe or Burundi* or Cambodia* or African or Chad or Chadian or Comoros or Cormoran or Congo* or Cote d'Ivoire or Ivorian or Eritrea* or Ethiopia* or Gambia or Ghana* or Guinea* or Guinea-Bissau* or Haiti* or Kenya* or Korea* or Kyrgyz or Kirghiz or Lao or Laotian or Liberia* or Madagascar or Malagasy or Malawi* or Mali or Malian or Mauritania* or Mozambi* or Burma or Burmese or Myanmar*

or

Nepal or Nepalese or Niger or Nigeri* or Pakistan* or Papua New Guinea* or Rwanda* or Sao Tome* or Principe or Senegal* or Sierra Leone* or Solomon Island* or Somali* or Tajik* or Tadzhik or Tanzania* or Togo or Togolese or Uganda* or Uzbek* or Vietnam* or Yemen* or Zambia* or Zimbabwe* or Albania* or Algeria* or Angola* or Armenia* or Azerbaijan* or Bhutan* or Bolivia* or Bosnia* or Herzegovina*

or

Cameroon* or Cape Verd* or China or Chinese or Colombia* or Congo* or Djibouti or Dominican or Ecuador* or Egypt* or El Salvador or Salvadoran or Georgia* or Guatemala* or Guyana or Hondura* or India or Indonesia* or Iran or Iranian or Iraq* or Jordan* or Kiribati or Lesotho or Mosotho or Basotho or Macedonia* or Maldives or Maldivan or Marshall Islands or Marshallese or Micronesia* or Moldova* or Mongolia* or Morocc* or Namibia* or Nicaragua*

or

Paraguay* or Peru or Peruvian or Philippines or Filipino or Samoa* or Sri Lanka* or Sudan or Sudanese or Swaziland or Swazi or Syria* or Thailand or Thai or Timor-Leste or Tonga* or Tunisia* or Turkmen* or Ukrain* or Vanuatu or Ni-Vanuatu or West Bank or Gaza or American Samoa or Argentin* or Belarus* or Belize* or Botswana or Motswana or Batswana or Brazil* or Bulgaria* or Chile or Chilean or Costa Rica*

or

Croatia or Croat or Cuba or Cuban or Dominica or Fiji* or Gabon or Gabonese or Grenada or Grenadian or Grenadan or Jamaica* or Kazakhstan* or Latvia* or Lebanon or Lebanese or Libya* or Lithuania* or Malaysia* or Mauritius or Mauritian or Mayotte or Mexic* or Montenegro or Palau* or Panama* or Poland or Pole or Polish or Romania* or Russian or Serbia* or Montenegrin or Seychell* or South Africa* or "Kitts and Nevis" or Kittian

or

Nevisian or "St. Lucia" or Saint Lucia* or "St. Vincent" or Saint Vincent or Grenadines or Suriname* or Turkey or Turk or Uruguay* or Venezuela*

)

exclude:

Immunology, Veterinary Science and Zoology

TI=(immunolog* or serolog*)

**7) CINAHL (4257)**

(MH "Immunization+") or (MH "Vaccines+") or vaccine* or vaccinat* or immuniz* or immunis*

AND

(MH "Developing Countries") or (develop* or low income or low-income or lower income or lower-income or middle income or middle-income) or Afghan* or Bangladesh* or Benin or Burkina Faso or Burkinabe or Burundi* or Cambodia* or African or Chad or Chadian or Comoros or Cormoran or Congo* or Cote d'Ivoire or Ivorian or Eritrea* or Ethiopia* or Gambia or Ghana* or Guinea* or Guinea-Bissau* or Haiti* or Kenya* or Korea* or Kyrgyz or Kirghiz or Lao or Laotian or Liberia* or Madagascar or Malagasy or Malawi* or Mali or Malian or Mauritania* or Mozambi* or Burma or Burmese or Myanmar* or Nepal or Nepalese or Niger or Nigeri* or Pakistan* or Papua New Guinea* or Rwanda* or Sao Tome* or Principe or Senegal* or Sierra Leone* or Solomon Island* or Somali* or Tajik* or Tadzhik or Tanzania* or Togo or Togolese or Uganda* or Uzbek* or Vietnam* or Yemen* or Zambia* or Zimbabwe* or Albania* or Algeria* or Angola* or Armenia* or Azerbaijan* or Bhutan* or Bolivia* or Bosnia* or Herzegovina* or Cameroon* or Cape Verd* or China or Chinese or Colombia* or Congo* or Djibouti or Dominican or Ecuador* or Egypt* or El Salvador or Salvadoran or Georgia* or Guatemala* or Guyana or Hondura* or India or Indonesia* or Iran or Iranian or Iraq* or Jordan* or Kiribati or Lesotho or Mosotho or Basotho or Macedonia* or Maldives or Maldivan or Marshall Islands or Marshallese or Micronesia* or Moldova* or Mongolia* or Morocc* or Namibia* or Nicaragua* or Paraguay* or Peru or Peruvian or Philippines or Filipino or Samoa* or Sri Lanka* or Sudan or Sudanese or Swaziland or Swazi or Syria* or Thailand or Thai or Timor-Leste or Tonga* or Tunisia* or Turkmen* or Ukrain* or Vanuatu or Ni-Vanuatu or West Bank or Gaza or American Samoa or Argentin* or Belarus* or Belize* or Botswana or Motswana or Batswana or Brazil* or Bulgaria* or Chile or Chilean or Costa Rica* or Croatia or Croat or Cuba or Cuban or Dominica or Fiji* or Gabon or Gabonese or Grenada or Grenadian or Grenadan or Jamaica* or Kazakhstan* or Latvia* or Lebanon or Lebanese or Libya* or Lithuania* or Malaysia* or Mauritius or Mauritian or Mayotte or Mexic* or Montenegro or Palau* or Panama* or Poland or Pole or Polish or Romania* or Russian or Serbia* or Montenegrin or Seychell* or South Africa* or "Kitts and Nevis" or Kittian or Nevisian or "St. Lucia" or Saint Lucia* or "St. Vincent" or Saint Vincent or Grenadines or Suriname* or Turkey or Turk or Uruguay* or Venezuela*

exclude:

animal studies

(MM "Veterinary Medicine") or (MM "Rabies") or (MM "Influenza, Avian") **or** (MM "Human Immunodeficiency Virus+") or TI immunolog* or TI serolog*

**3. SOP for abstract review and quality assessment**

**A. Review of titles and abstracts**

All titles and abstracts were reviewed in duplicate. Each title/abstract was categorized into one of the three categories:

1. Highly relevant
2. Possibly relevant
3. Not relevant

These categories are defined as follows:

1. *Highly relevant* – titles that included one or a combination of the following terms:
   - - - 1. Routine immunization
         2. Infant (or child) health service utilization
         3. Immunization coverage (or survey)
         4. Coverage of measles (or other specific EPI vaccination)

If the title did not include these terms, and the abstract, if available, for highly relevant articles had to mention routine immunization and reasons/risk factors related to a child vaccination status.

In some cases, these terms were included in the title, but the article was still excluded. These exceptions are outlined below in “Not relevant” section.

2) *Possibly relevant* – titles did not include above, but included broader reference immunization strategies or immunization initiatives in either the title or abstract were placed in this category.

3) *Not relevant* - if neither the title nor abstract included reference to routine immunization, the articles were excluded as not relevant.

This included the following articles

1. Articles investigating immunogenicity of a EPI vaccine (and other serological, genetic investigations)
2. Articles investigating non-EPI related immunization, such as HIV, Malaria, HPV, Influenza, etc.
3. Articles describing adult vaccinations
4. Articles describing global immunization policy (such as optimal schedules, etc.)
5. Articles describing cancers, anti-smoking initiatives, etc.

Also excluded were articles describing coverage and impact of a mass campaign or general articles describing the epidemiology of VPDs in a country or region.

**B. Review of Highly Relevant articles**

1) Every article will be initially reviewed by two persons to assess overall relevancy. If the article appears relevant to both reviewers, then this article will be forwarded to two persons.

2) For each article, one reviewer will be the primary reviewer and one reviewer will be the secondary reviewer.

3) Each reviewer will complete the abstraction form (specific instructions provided), resulting in two completed abstraction forms for each article.

4) Articles not in a WHO language, not indicating reasons/factors for being under/not vaccinated, not an EPI vaccination, or not a Peer-reviewed journal – will be excluded. We will attempt to identify non-peer reviewed articles in advance. However, this may, on some occasions be determined by the reviewers.

5) Abstraction forms will be reviewed for any differences, which will be discussed and reconciled, if necessary.

6) After reconciliation, data will be entered in the text database.

7) Each step will be tracked in a excel spreadsheet to assess progress including the number of relevant articles published each year, number of non-peer reviewed articles (to be shared with Immunization Basics), outstanding forms, and status of data entry.

**C. Quality assessment**

Quality of Study

Articles identified as highly relevant are assessed for study/project quality – according to the following 10 criteria. Articles must have a score of 70% (or meet at least 7 of the 10 criteria below) to be included in the final analysis.

1) Study question/hypothesis/purpose of the project defined

2) Target population defined

3) Methods included

4) Recruitment/sampling scheme described

5) Analyses described

6) Source of vaccination information

7) Data/results presented

8) Findings compared to other studies

9) Limitations addressed

10) Major conclusion described

**D. Codes for title, abstract and full text review**

**Criteria for inclusion:**

Papers to be included in the review will focus on the explaining **WHY** and **HOW** identified barriers limit access to immunization services. Specifically we are looking for the ***gender based reasons*** specific barriers limit access to immunization. See framework for list of barriers that may have a gender dimension. Papers must focus on general population based immunization programmes (targeted campaigns will also be included and coded with c during abstract review) that provide vaccination on **Diptheria, Pertusis, Tetnus (DPT), tetanus toxoid vaccine (TT), oral polio vaccine (OPV) , Bacillius-Calmette.Guérin (CPG/BCG), and Measles, Mumps and Rubella (MMR).**

**Criteria for exclusion:**

Papers to be excluded focus on the **wrong vaccine** (e.g. Flu (any type), Cholera, Human Papiloma Virus (HPV), HIV, Hepatitis A-D) the **wrong target group** (e.g. other than children under 5 (59months)), **wrong country or region** (e.g. developed countries, high income countries => refer to World Bank Classification), or **wrong issue** (e.g. vaccine schedules, incomplete coverage (received one dose but not two), health system factors that we excluded (i.e. supply chain, insurance issues, procurement and distributions, etc => refer to gender analysis framework).

**Title review:**

| **Location** | **Marking** | **Reason** |
| --- | --- | --- |
|  |  |  |
| Custom 1 | -none- | Title out |
| Custom 1 | tin | Title in |

**Abstract review (with “tin”):**

| **Location** | **Marking** | **Reason** |
| --- | --- | --- |
|  |  |  |
| Custom 1 | [tin] abin | Correct topic (qualitative or quantitative) |
| Custom 2 | qual | ABIN for qualitative review |
| Custom 2 | quant | ABIN survey data |
| Custom 1 | [tin] abx 1 | Abstract out - Wrong topic |
| Custom 1 | [tin] abx 2 | Abstract out - Correct topic; wrong region |
| Custom 1 | [tin] abx 3 | Abstract out - Correct topic; wrong design (e.g. letters, not original research, clinical trial, etc) |
| Custom 1 | [tin] int | Potentially interesting article for discussion |
| Custom 1 | [tin] noab | Abstract not available |
| Custom 2 | [qual/quant] C | Campaign |

**Full text review (with “abin”):**

| **Location** | **Marking** | **Reason** |
| --- | --- | --- |
|  |  |  |
| Custom 1 | [tin] [abin] ftin | Correct topic (qualitative or quantitative) |
| Custom 2 | qual | FTIN for qualitative review |
| Custom 2 | quant | FTIN survey data |
| Custom 1 | [tin] [abin] ftx 1 | Full text out - Wrong topic |
| Custom 1 | [tin] [abin] ftx 2 | Full text out - Correct topic; wrong region |
| Custom 1 | [tin] [abin] ftx 3 | Full text out - Correct topic; wrong design (e.g. letters, not original research, clinical trial, etc) |
| Custom 1 | [tin] [abin] int | Potentially interesting article for discussion |
| Custom 1 | ftna | Full text not available |

**Full text review for quality (with ftin/qual only):**

| **Location** | **Marking** | **Reason** |
| --- | --- | --- |
|  |  |  |
| Custom 3 | 1 | FTIN AND contains original qualitative data |
| Custom 3 | 2 | FTIN AND DOES NOT contain sufficient original qualitative data |
| Custom 3 | 3 | FTIN AND poor methods (e.g. the size, methods used, or approach is insufficiently scientific (internal sample, biased, too small sample, etc) ) |
| Custom 1 | [tin] [abin] [ftin] [qual] int | Potentially interesting article for discussion |

***NB. Full text extraction will be done with extraction sheets and coding in ATLAS ti software.***
