# Supplementary material for: Gender Determinants of Vaccination Status in Children: Evidence from a Meta-Ethnographic Systematic Review
Source: PLoS One. 2015 Aug 28;10(8):e0135222. doi: 10.1371/journal.pone.0135222 (PMC4552892; doi:10.1371/journal.pone.0135222)
Supplement: S2 Annex — (DOC) [file pone.0135222.s003.doc]

**S2 Annex -** Study characteristics table

| # | Author(s) | Year | Country | Urban /rural | Sample subgroup | Method of data collection | IDIs | FGDs | Study objective |
| --- | --- | --- | --- | --- | --- | --- | --- | --- | --- |
| 1 | Azevedo | 1991 | Cameroon | rural | Opinion leaders and individuals of the community | FGDs |  | 29 | To ascertain the attitudes, beliefs, and practices associated with child mortality and usage of modern health care centres |
| 2 | Bastien | 1995 | Bolivia | rural | Community members | Open ended interviews and participant observation | n.a. | n.a. | To analyze cultural beliefs and practices in order to understand how people in three cultural areas in Bolivia (Aymara, Quechua and Tupi-Guarani) think about NNT and tetanus toxoid |
| 3 | Babirye | 2011 | Uganda | urban | Mothers | Open ended interviews | 8 | 9 | To examine influences on childhood immunisation behaviour using the attitude-social influence-self efficacy model |
| 4 | Berhanel | 2000 | Ethiopia | rural | Health workers and community members; Mothers of children <5 years | In depth interviews (IDIs); KI interviews, FGDs; Document review; Observations | n.a. | 96 | To understand social, cultural. political and economic factors that influence the efficiency and effectiveness of immunization Programs |
| 5 | Bisht | 2000 | India |  | Mothers of children between the ages of 0 to 2 years | IDIs | 205 |  | To understand the various perceptions of the causation of measles and the quest for therapy in different socio-cultural contexts |
| 6 | Braka | 2010 | Uganda | rural | Mothers of children <5 years; Community leaders | IDIs; FGDs; Non-formal interactions (n=156); Observations. | 25 | 12 | To identify community sources of information on immunization; to determine caretakers’ knowledge and concerns of immunization; to determine the influence of vaccine concerns on caretakers’ decisions to vaccinate; to understand perceptions and experience of HCWs and policy makers about vaccine safety and adverse events |
| 7 | Chaturvedi | 2009 | India | rural | Mothers of children <5 years; Community leaders | IDIs; FGDs; Non-formal interactions (n=156); Observations. | 43 | 12 | To gain an insight into the phenomenon of social resistance and rumours against pulse polio campaign. |
| 8 | Coreil | 1994 | Haiti | urban | Mothers of preschool children and health care providers | FGDs; Natural groups interviews | 4 | 26 | To understand the social context of immunization seeking behaviour and to help develop a culturally valid survey instrument for use in a subsequent case-control study |

**Annex I: Study characteristics table (continued)**

| **#** | **Author(s)** | | **Year** | **Country** | **Urban /rural** | **Sample subgroup** | **Method of data collection** | **IDIs** | **FGDs** | **Study objective** |
| --- | --- | --- | --- | --- | --- | --- | --- | --- | --- | --- |
| 9 | Dasgupta | 2008 | | India | urban/rural | Mothers of children <5 years; Community leaders | Qualitative. Rapid appraisal procedures (RAP) | 43 |  | To understand the perceptions and likely determinants that facilitate or act as barriers in implementing additional strategies for polio eradication |
| 10 | Eng | 1991 | | Togo | rural | Mothers | FGDs |  | 110 | To investigate the lack of acceptance of childhood immunisation from a social science perspective |
| 11 | Fassin | 1986 | | Senegal | urban/rural | Mothers of children <5 years | Semi structured interviews | 100 |  | To investigate the relation between social groups in rural and urban areas |
| 12 | Helman | 2004 | | Transkei | rural | Caretakers of children <5 years | Semi-structured Interviews; FGD | 60 | 17 | To examine perceptions of childhood illnesses, and the role of immunisation in preventing them, among caretakers of young children |
| 13 | Li | 2004 | | China | rural | Mothers of children born in the 3 years prior to the study | FGDs |  |  | To test the general hypothesis that gender inequality and the state’s family planning policy have a significant influence on Maternal and childcare utilization |
| 14 | Mavimbe | 2006 | | Mozambique | urban | Official district Directors | Semi-structured interviews; Participant observation; Secondary data collection | 14 |  | To ascertain the construction of immunization coverage and how they implement the desired program strategies in order to improve the health status of the region |
| 15 | Odebiyi | 1982 | | Nigeria |  | Older mothers | IDIs | 200 |  | To assess the mothers' awareness of the seriousness of the disease and their knowledge of its prevention |
| 16 | Odebiyi | 1993 | | Kenya | rural | Mothers attending the clinic | IDIs | 184 |  | To examine the role of the women in the expanded program of immunization (EPI) |
| 17 | Oluwadare | 2009 | | Nigeria | urban/rural | Mothers; Health workers; Community leaders | FGDs; KI interviews | n.a. | n.a. | To explain the intractable plummeting trend of immunisation in Nigeria and in Ekiti State as a case study |

**Annex I: Study characteristics table (continued)**

| **#** | **Author(s)** | **Year** | **Country** | **Urban /rural** | **Sample subgroup** | **Method of data collection** | **IDIs** | **FGDs** | **Study objective** |
| --- | --- | --- | --- | --- | --- | --- | --- | --- | --- |
| 18 | Pool | 2006 | Mozambique | urban/rural | Mothers/caretakers of infants | IDIs and semi-structured interviews, Participant observation | 308 |  | To describe attitudes to the expanded programme on immunization (EPI) and intermittent preventive treatment in infants and perceptions of the relationships between them |
| 19 | Renne | 2006 | Nigeria | urban/rural | Parents; students (without children) who had polio; immunization workers and local government health officials members | IDIs; Participant observation | 32 |  | To examine the reasons for the difficulties in eradicating polio from the perspective of community members |

| 20 | Schwarz | 2009 | Gabon |  | Mothers of children <5 years | IDIs; informal conversations;  observations | 40 |  | To explore attitudes of mothers towards childhood vaccinations and reasons for non-attendance and non-adherence to mother–child clinics (MCCs) |
| --- | --- | --- | --- | --- | --- | --- | --- | --- | --- |
| 21 | Suresh | 2000 | India | urban/rural | Children between 0-59 months | Semi-structured interviews | 20 |  | To validate the reported coverage for 1999-2000 |
| 22 | Tadesse | 2009 | Ethiopia | rural | Community representatives in Wonago district | FGDs; IDIs | 22 | 6 x ? | To explore factors associated with child immunization |
| 23 | Topuzoglu | 2007 | Turkey | suburban | Mothers of children <5 years | FGDs; IDIs | 2 | 8 x ? | To understand the behaviours of mothers concerning the immunization of their children, the decision-making process, the perceived barriers, and the enabling factors to access the services |
| 24 | Uddin | 2010 | Bangladesh | urban | Mothers of children aged 12–23 months and service providers | FGDs; IDIs; Participant observations | 16 | 8 x ? | To assess the impact of an EPI intervention package |
| 25 | Unisa | 2006 | Jharkhand / Rajasthan |  | Programme officials and community members | FGDs; IDIs | 704 | 30 | To explore the problems fro supply side of the immunisation services and to examine the demand for immunisation services, knowledge and utilization of the different typed of vaccines by the community |
