# Supplementary material for: Gender Determinants of Vaccination Status in Children: Evidence from a Meta-Ethnographic Systematic Review
Source: PLoS One. 2015 Aug 28;10(8):e0135222. doi: 10.1371/journal.pone.0135222 (PMC4552892; doi:10.1371/journal.pone.0135222)
Supplement: S3 Annex — (DOC) [file pone.0135222.s004.doc]

**S3 Annex – Translation table**

| **3rd order labels** | **3rd order constructs** | **2nd order constructs** | **Gendered factor** | **Source papers** |
| --- | --- | --- | --- | --- |
| **1. Access** |  |  |  |  |
| ***a) Availability*** |  |  |  |  |
| **Supply and infrastructure constraints** | *Supply failures limit effectiveness of immunization programmes* | Shortage in supply of vaccines, cold chain capacity, and electricity; no health cards available | no | Azevedo 1991; Bernahel, 2000, Eng 1991; Fassin 1986; Helman 2004; Odebiyi 1993; Oluwadare 2009¸ Tadesse 2009, Schwarz 2009; Unisa 2006; |
| **Human resource constraints** | *Lack of human resources, and working condition limit motivation* | Human resource shortages due to lack of qualified personnel, financial constraints, disorganisation | no | Oluwadare 2009¸ Tadesse 2009; Unisa 2006, Bernahel, 2000, Suresh 2000, Odebiyi 1993 |
|  | *Missed opportunities to provide vaccination* | Providers have poor technical skills, are poorly trained, and may give incorrect information as regards vaccination schemes | no | Bastien 1995; Bernahel 2000; Topuzoglu 2007 |
|  | *Incentives inadequate* | Targets for health professionals may lead to low quality management and poor reporting | no | Dasgupta 2008 |
| ***b) Accessibility*** |  |  |  |  |
| **Limited outreach of services** | *Failure to reach rural and poor communities* | Unpredictable location of post; lack of health posts | no | Coreil 1994; Helman 2004; Odebiyi 1993; Oluwadare 2009; Tadesse 2009; Uddin 2010; Unisa 2006; Bernahel 2000; Schwarz 2009 |
|  |  | Importance of minimizing transportation barriers | implicit | Coreil 1994; Fassin 1986; Helman 2004; Odebiyi 1993; Oluwadare 2009; Schwarz 2009; Tadesse 2009; Topuzoglu 2007; Unisa 2006; Bernahel 2000 |
|  |  | Mobile units, home visits and community support groups | implicit | Azevedo 1991; Bernahel 2000; Helman 2004; Oluwadare 2009; Tadesse 2009; Uddin 2010 |
| ***c) Affordability*** |  |  |  |  |
| **Costs of service** | *Direct and indirect costs limits utilization* | Direct and indirect costs, e.g. transport costs; informal fees | implicit | Azevedo1991; Coreil 1994; Helman 2004; Oluwadare 2009; Tadesse 2009; Topuzoglu 2007; Bernahel 2000; Schwarz 2009; Fassin 1986 |
| **Entitlement to resources** | *Cost and resource allocation* | Constraints to mobilize funds for direct and indirect costs of vaccination | yes | Azevedo 1991; Bisht 2000; Coreil 1994; Olu-wadare 2009; Schwarz 2009; Topuzoglu 2007 |
|  |  | Control over resources, bargaining power | yes | Coreil 1994; Li 2004; Renne 2006; Pool 2006; Oluwadare 2009; Topuzoglu 2007; Schwarz 09 |
|  |  | Intra-family power relations | yes | Bastien 1995; Eng 1991; Pool 2006; Olu-wadare 2009; Tadesse 2009, Topuzoglu, 2007 |
| **Social inequality** | *Social resources* | Marginalisation of the poor | implicit | Coreil 1994; Chaturvedi 2009; Fassin 1986; Odebiyi 1993; Oluwadare 2009; Topuzoglu 07 |
|  |  | Lack of social support for women | yes | Coreil 1994; Uddin 2010 |
|  | *Poverty and shame* | Lack of resources; illiteracy | yes | Chaturvedi 2009, Coreil 1994; Odebiyi 1982 |
|  |  | Shame/ public discrimination linked to poverty | implicit | Coreil 1994; Fassin 1986; Schwarz 2009 |
| ***d) Accommodation*** | |  |  |  |
| **Lack of responsiveness of service** | *Responsiveness of services to the actual needs of clients* | Provider views on accommodation barriers disregard client' views | implicit | Coreil 1994; Suresh 2000 |
|  | *Failure to offer services to working parents* | Extension of service hours to reach working mothers | implicit | Bernahel, 2000; Uddin 2010 |
|  | *Programme design can accommodate cultural and ethnic beliefs* | Ethnicity of user and provider may be linked to social and language barriers | implicit | Bastien 1995 |
|  | *Vertical immunization programs counteract local priority setting of healthcare needs* | Prioritizing vertical immunization programmes over PHC is not responsive to local needs | no | Chaturvedi 2009; Helman 2004; Mavimbe 2006; Oluwadare 2009, Bernahel 2000; Schwarz 2009 |
|  |  | Focus on vertical programme can raise suspicion of hidden motivations | no | Renne 2006; Dasgupta 2008; Bernahel 2000 |
|  |  | Healthcare providers should be involved in setting priorities and goals in order to better meet local needs | no | Mavimbe 2006; Bernahel 2000; Oluwadare 2009; Unisa 2006; Schwarz 2009 |
| ***e) Acceptability*** |  |  |  |  |
| **Limited availability of services** | *Failure to offer services to working mothers* | Extension of service hours to reach working mothers | yes | Bernahel, 2000; Uddin 2010 |
| **Authoritative health system** | *Limited informed choice by user* | Dependency on clinic and fear of reprisal makes clients accept low quality of care and disrespectful treatment | implicit | Pool 2006, Bernahel, 2000 |
| **Discriminatory provider-user-interaction** | *Patronizing counselling* | Lack of privacy at health posts results in public humiliation of deprived mothers | yes | Coreil 1994; Helman 2004; Oluwadare 2009; Tadesse 2009; Topuzoglu 2007; Bernahel 2000; Schwarz 2009 |
|  |  | Providers treat caretakers, particularly mothers, rudely, demonstrating disrespect for their time, effort or interests. | yes | Bastien 1995; Schwarz 2009; Coreil 1994; Helman 2004; Topuzoglu 2007 |
|  |  | Providers do not adequately explain how vaccinations work | implicit | Bastien 1995; Helman 2004; Topuzoglu 2007 |
|  |  | Providers blame mothers as not understanding the importance of vaccinations | yes | Bernahel 2000; Topuzoglu 2007 |
| **Gender norms and obligations** | *Feminized tasks and competing obligations* | Competing priorities to meet everyday needs | yes | Chaturvedi 2009, Coreil 1994; Schwarz 2009; Topuzoglu 2007 |
|  |  | Productive role/competing priorities | yes | Coreil1994; Oluwadare 2009; Schwarz 2009 |
|  |  | Domestic role/competing priorities | yes | Coreil1994; Li 2004; Topuzoglu 2007; Schwarz, 2009 |
|  |  | Maternal role/competing priorities | yes | Bastien 1995; Coreil 1994; Li 2004; Odebiyi 1993; Topuzoglu 2007 |
| ***f) Agency*** |  |  |  |  |
| **Limited agency** | *Female isolation and lack of autonomy* | Burden to represent the household | yes | Coreil 1994; Oluwadare 2009; Topuzoglu 2007 |
|  |  | Women’s subordinate status/role in society; lack of female autonomy | yes | Bastien 1995, Coreil 1994; Li 2004, Pool 2006, Topuzoglu 2007 |
|  |  | Men’s higher status in society | yes | Oluwadare 2009, Tadesse 2009, Topuzoglu 2007 |
|  |  | Men’s limited participation in childcare | yes | Bastien 1995 |
|  | *Son preference* | Son preference | yes | Li 2004, Topuzoglu 2007 |
|  | *Psychosocial distress* | Physical overload | no | Coreil1994; Oluwadare 2009; Schwarz 2009 |
|  |  | Psychological overload | no | Coreil 1994; Topuzoglu 2007 |
|  |  | Intimate partner violence | yes | Coreil 1994; Topuzoglu 2007 |
|  |  | Gendered blame if child health status is inadequate | yes | Bastien 1995 |
| **Discrimination of women and girls** | *Girls and women’s social status linked with immunization* | Son preference linked to mother’s status in society; determinant of immunization status | yes | Li 2004 |
|  |  | Government emphasis on status of women created support for healthy families | yes | Nair 2007 |
| **2. Demand for vaccination services** | |  |  |  |
| ***a) Literacy*** |  |  |  |  |
| **General education and literacy** | *Impact of education levels and literacy on immunization status* | Literacy is correlated with vaccine acceptance/demand | implicit | Azevedo 1991, Bastien 1995, Eng 1991, Li 2004, Odebiyi 1982, Oluwadare 2009, Renne 2006, Topuzoglu 2007 |
|  |  | YET education has no impact on beliefs in supernatural causes of diseases | no | Azevedo 1991; Odebiyi 1982 |
|  | *Unequal access to education* | Gender inequality in education | yes | Bastien 1995, Li 2004 |
|  |  | Differentials between religious groups in access to education | no | Oluwadare 2009, Renne 2006 |
|  |  | Geographic inequality in access to education | no | Bastien 1995 |
| **Health literacy** | *Unequal access to health information* | Problem of access to information related to vaccination | implicit | Azevedo 1991, Helman 2004, Odebiyi 1982, Tadesse 2009. Eng 1991 |
|  | *Quality of health information* | Information mainly provided by health personnel, however often poorly trained | no | Bastien 1995, Bisht 2000, Coreil 1994, Schwarz 2009, Fassin 1986, Topuzoglu 2007 |
|  |  | The problem of naming the conditions | no | Bastien 1995; Bernahel 2000; Bisht 2000; Renne 2006; Fassin 1986 |
|  | *Experiential knowledge and legitimacy of information* | Health seeking as a process that builds meaning through experience important for women | yes | Bisht 2000; Bernahel 2000; Tadesse 2009; Fassin 1985; Suresh 2000 |
|  |  | Local sources/carriers of knowledge may have more legitimacy than health professionals, e.g. older women, community leaders | yes | Bisht 2000, Eng 1991, Oluwadare 2009, Renne 2006, Suresh 2000 |
|  |  | Preference for traditional medicine linked to poor treatment by western providers | no | Bisht 2000 |
|  |  | *A posteriori* knowledge: empirical health knowledge acquired through personal or proxy experience | no | Bernahel 2000, Bisht 2000, Coreil 1994, Fassin 1986, Odebiyi 1982, Tadesse 2009, Topuzoglu 2007 |
| ***b) Communication*** | |  |  |  |
| **Communication in vaccination campaigns** | *Insufficient information campaigns* | Need for more and better information campaigns; non-vertical approaches | no | Azevedo 1991, Fassin 1986 Bastian 1995, Renne 2006, Oluwadare 2009 |
|  | *Lack of “cross-cultural” communication* | Respect local aetiologies, translating ideas may facilitate understanding of the concept of immunization | no | Bastien 1995 |
|  | *Feminization of information* | Need to address men who are often the decision-makers | yes | Pool 2006 |
| ***c) Knowledge claims*** | |  |  |  |
| **Knowledge claims and experiental knowledge** | *Evolution of knowledge systems* | Traditional knowledge itself includes elements of external pre-colonial and colonial influences; no pure 'traditional medicine' | no | Bastien 1995 |
|  |  | Incorporation of different knowledge systems; reinterpretation of knowledge when imported | no | Bernahel 2000, Bisht 2000, Eng 1991; Pool 2006 |
|  |  | Health centre main source of vaccine-related knowledge | no | Bastien 1995, Oluwadare 2009, Suresh 2000 |
|  | *Knowledge claims of biomedicine are challenged* | Persistence of culturally embedded local knowledge despite knowledge claims of biomedical system | no | Azevedo 1991; Bisht 2000 |
| ***d) Local healing concepts*** | |  |  |  |
| **Local illness perceptions of vaccine preventable diseases** | *Supranatural causes of illness* | Supernatural illness causation important in community | no | Azevedo 1991, Bastien 1995, Bernahel 2000, Bisht 2000, Eng 1991, Fassin 1986, Renne 2006, Odebiyi 1982, Tadesse 2009 |
|  |  | Dramatic symptoms must be caused by supernatural forces | no | Bastien 1995 |
|  |  | Wind, vapours, hot and cold as ‘natural’ origin of illness | no | Bastien 1995, Bernahel 2000, Bisht 2000, Coreil 1994, Fassin 1986 |
|  |  | Germ theory rare in local causal explanations | no | Azevedo 1991; Helman 2004; Odebiyi 1982 |
|  |  | Externalised danger: construction of otherness as the space of contagion” | no | Bisht 2000 |
|  | *Religion affects acceptance of knowledge claims* | Impact of religion on attitudes and values | no | Azevedo 1991; Bastian 1995; Bisht 2000; Renne 2006; Oluwadare 2009 |
|  | *Witchcraft/sorcery as social dimension of illness* | Behavioural causes, moral transgressions as cause of illness | yes | Azevedo 1991, Bastien 1995, Bisht 2000, Fassin 1986 |
|  |  | Secrecy of witchcraft; addressing social malfunctioning | yes | Bastien 1995, Fassin 1986 |
|  |  | Witchcraft as a gendered power discourse | yes | Bastien 1995 |
|  | *Local taxonomy and biomedical symptoms* | Local symptomatology coincides with biomedical concept | no | Bisht 2000, Fassin 1986 |
|  |  | Local symptomatology doesn’t coincide with biomedical concept | no | Bastien 1995, Bernahel 2000; Bisht 2000 |
|  | *Knowledge of Vaccine Preventable Disease (VPDs)* | Differential knowledge of VPDs | no | Bernahel 2000, Coreil 1994, Helman 2004 |
| **Concepts of treatment and prevention** | *Perceived effectiveness of treatment options* | Using both western medicine and traditional medicine, also combining vaccination and traditional protection | no | Azevedo 1991, Bastian1995, Bernahel 2000, Bisht 2000, Odebiyi 1982, Renne 2006, Tadesse 2009 |
|  |  | Illness-specific treatments e.g. in case of supernatural causes, with implications for prevention | no | Azevedo 1991, Bastian1995, Bernahel 2000, Bisht 2000, Renne 2006, Tadesse 2009 |
|  |  | Healing through divine intervention, with implications for prevention | no | Azevedo 1991, Bastien 1995, Bisht 2000, Odebiyi 1982, Oluwadare 2009 |
|  |  | Preference for home treatment | implicit | Fassin 1986, Bernahel 2000, Bisht 2000 |
|  |  | Preference for Western medicine | no | Helman 2004 |
|  | *Treatment through traditional practices* | Non-therapeutic practices: care, spiritual protection | no | Bernahel 2000, Fassin 1986, Odebiyi 1982 |
|  |  | Local therapeutic practices may be harmful (e.g. food taboos) | no | Azevedo 1991, Bastien 1995, Bernahel 2000, Fassin 1986 |
|  | *Concepts of prevention and protection* | Understanding prevention as different from traditional protection | no | Bernahel 2000, Bastien 1995, Bisht 2000, Eng 1991, Fassin, 1986, Odebiyi 1982, Pool 2006 |
|  |  | Protection is essential part of maternal care | yes | Bisht 2000 |
|  |  | Children's diseases understood as not preventable | no | Bastien 1995; Bernahel 2000; Bisht 2000; Fassin 1986; Odebiyi 1982; Tadesse 2009 |
|  | *Knowledge of vaccine and immunization* | Awareness of the necessity of immunization | no | Bernahel 2000, Azevedo 1991, Eng 1991, Helman 2004, Oluwadare 2009, Tadesse 2009, Topuzoglu 2007, Unisa 2006 |
|  |  | Misconceptions: confusion between prevention and cure or protection | no | Bernahel 2000, Bisht 2000, Coreil 1994, Eng 1991, Fassin 1986, Helman 2004, Odebiyi 1982, Oluwadare 2009, Pool 2006, Schwarz 2009, Tadesse 2009, Topuzoglu 2007, Unisa 2006 |
|  |  | Not understanding immunization, e.g. not relating vaccines to specific diseases | no | Bisht 2000, Coreil 1994, Fassin 1986, Helman 2004, Odebiyi 1982, Oluwadare 2009, Pool 2006, Tadesse 2009, Topuzoglu 2007, Unisa 2006 |
|  |  | Not knowing the regimen (planning of vaccinations) | no | Coreil 1994, Eng 1991, Suresh 2000, Topuzoglu 2007, Uddin 2010, Unisa 2006 |
| ***3. Trust in vaccination services*** | |  |  |  |
| **Immunisation program misused for political manipulations** | *Resistance to vaccination* | Immunisation programmes are politicized in order to manipulate people, mainly through rumours that vaccines cause sterility, which are spread by religious leaders to create resistance/ power shift | implicit | Bastien 1995; Chaturvedi 2009; Oluwadare 2009; Pool 2006; Renne 2006 |
|  |  | Fear of attacks on fertility/fecundity and fear of genocide are rooted in experiences with coercive population control and are often associated with female sorcery/witchcraft | yes | Bastien 1995; Dasgupta 2008; Renne 2006; Topuzoglu 2007 |
|  |  | Resistance to vaccination as social resistance to outsiders | no | Chaturvedi 2009; Pool 2006 |
|  |  | Resistance to vaccination as social resistance by caste/class | no | Fassin 1986; Odebiyi 1982; Pool 2006 |
|  |  | Suspicion of self interest to promote biomedicine (conspiration) | no | Renne 2006 |
|  |  | Suspicion greater among vulnerable populations and marginalised groups | no | Oluwadare 2009; Pool 2006; Chaturvedi 2009 |
| **Governance** | *Government priorities fail to meet local needs* | Government not trusted because they don’t meet expressed needs such as PHC for children but invest in vaccines | no | Azevedo 1991; Renne 2006 |
|  | *Coercive governance and population control* | Colonial style administration of immunisation programs | no | Bastien 1995; Dasgupta 2008; Pool 2006 |
|  |  | Coercive practices of government to increase coverage of immunisation | no | Chaturvedi 2009 |
|  |  | Use of incentives distorting the system and is ripe for bargaining by the people | no | Dasgupta 2008; Fassin 1986 |
|  |  | Historical policy context that emphasises gender roles and social control | yes | Li 2004; Topuzoglu 2007 |
|  |  | Fear of acting counter to the rules or governmental authority | no | Pool 2006; Schwarz 2009 |
|  |  | Reduction in donors funds impacts on services | no | Bernahel 2000 |
